# Nano-laminography with a transmission X-ray microscope

**DOI:** 10.1107/S1600577525007234

**Published:** 2025-09-10

**Authors:** Viktor Nikitin, Alberto Mittone, Samuel J. Clark, Kamel Fezzaa, Michael Wojcik, Alex Deriy, Sunil Bean, Francesco De Carlo

**Affiliations:** a Advanced Photon Source Argonne National Laboratory Lemont IL60439 USA; Paul Scherrer Institut, Switzerland

**Keywords:** laminography, transmission X-ray microscope, nano-tomography

## Abstract

Nano-laminography combines the penetrating power of hard X-rays with a tilted rotational geometry to deliver high-resolution, three-dimensional images of laterally extended, flat specimens that are otherwise incompatible with, or difficult to image using, conventional nano-tomography. In this work, we demonstrate a full-field, X-ray nano-laminography system implemented with the transmission X-ray microscope at beamline 32-ID of the upgraded Advanced Photon Source at Argonne National Laboratory, USA. By rotating the sample around an axis inclined by 20° to the incident beam, the technique minimizes the long optical path lengths that would otherwise generate excessive artifacts when planar samples are imaged edge-on. The efficiency of the technique is demonstrated with 50 nm spatial resolution and minute-scale temporal resolution 3D imaging of a planar integrated circuit sample and targeted imaging of an individual particle within a powder sample, where mounting procedures are typically challenging in regular nano-tomography. The sample mounting strategy, data acquisition, and reconstruction method will also be discussed.

## Abstract 2

A full-field, X-ray nano-laminography system implemented with the transmission X-ray microscope at beamline 32-ID of the upgraded Advanced Photon Source at Argonne National Laboratory is demonstrated.

## 1. Introduction

The transmission X-ray microscope (TXM) has proven to be an efficient tool for *ex situ* and *in situ* 3D characterization of specimens in the nano-resolution regime across many scientific applications, including battery research (Spence *et al.*, 2021 ▸; Lin *et al.*, 2020 ▸; Hallot *et al.*, 2022 ▸), microelectronics (Wang *et al.*, 2002 ▸; Kutukova *et al.*, 2018 ▸), and biomedical imaging (Mizutani *et al.*, 2019 ▸; Nikitin *et al.*, 2021 ▸; Albers *et al.*, 2024 ▸; Bonnin *et al.*, 2024 ▸; Flenner *et al.*, 2022 ▸). In a typical TXM, an X-ray beam is focused using an X-ray condenser to a small focal spot where the sample is placed; the beam then diverges and passes through a Fresnel zone plate (FZP) acting as an X-ray objective lens; after the FZP the beam propagates to the detection system located several meters downstream. The TXM is a high-throughput full-field imaging instrument, which allows one to perform a full tomography scan on a minute scale at a resolution of around 30–60 nm (Ge *et al.*, 2018 ▸) and on an hour scale to deliver a resolution of down to 10 nm (De Andrade *et al.*, 2021 ▸). So far, such time scales have not been demonstrated by actively developed scanning techniques such as 3D ptychography which delivers 4–20 nm resolution phase-contrast imaging but currently requires tens of hours to days of scanning time (Holler *et al.*, 2017 ▸; Deng *et al.*, 2019 ▸; Aidukas *et al.*, 2024 ▸), although data collection times are expected to be dramatically reduced at recently upgraded fourth-generation facilities like of the Advanced Photon Source (APS) and European Synchrotron Radiation Facility (ESRF). At the same time, 3D reconstruction of TXM data can be performed in almost real time using packages like *TomocuPy* (Nikitin, 2023 ▸) and *TomoStream* (Nikitin *et al.*, 2022 ▸), which provide a great advantage for fine tuning the experimental parameters. Therefore, TXM represents a unique tool for dynamic *in situ* experiments or whenever a large number of samples need to be imaged to obtain acceptable statistics on the material structure. This capability can be further extended to study properties at the macroscale.

As with any other X-ray nano-imaging technique, TXM presents challenges in scanning irregularly shaped, large samples and in sample mounting procedures. A general limitation for samples imaged with a TXM is the thickness that X-rays need to traverse, along with constraints related to the limited depth of field of the zone plate. For instance, a 50 nm zone plate at 8 keV X-ray energy has a depth of field of approximately 50 µm. This means that imaging and processing samples thicker than 50 µm may experience blurring and a loss of contrast. On the other hand, working with pillar-shaped samples that must be less than 50 µm thick in the X-ray beam propagation direction for a full sample rotation is challenging, as such samples typically need to be mounted on a pin. This procedure is quite delicate, involving the use of a micro-manipulator, high-resolution microscope, and epoxy or another adhesive to attach the sample to the tip of the pin. Furthermore, due to the limited depth of field of optical microscopes, it is quite common for the sample to end up at the edge of the pin tip, which can block the beam and severely compromise tomographic imaging.

In addition to the challenges related to sample thickness and mounting procedures, the preparation of environmental or *operando* cells to study micrometre-sized samples with TXM is also difficult. As an example, studying *operando* battery cycling processes requires coin or pouch cells with connected wires. Such cells result in a large range of blocked angles when scanning in the conventional tomography regime, and are therefore typically used for radiography, *i.e.* 2D imaging without rotation (Nelson *et al.*, 2012 ▸). Another example is electrical/mechanical tests of integrated circuits or other microelectronics samples. In general, it is not a problem to perform tomography on a very thin, flat circuit that has been cut to 200–300 µm in both lateral dimensions. However, such samples may not describe the actual *operando* behavior of a 5 mm chip. Scanning with additional devices connected to the sample borders may result in large 30–40° angular blockage, as well as radiation/heating damage to these devices since they may appear in the beam path at some angles. To the best of our knowledge, no dynamic nano-imaging studies of integrated circuits have been reported to date.

**Figure 1:**
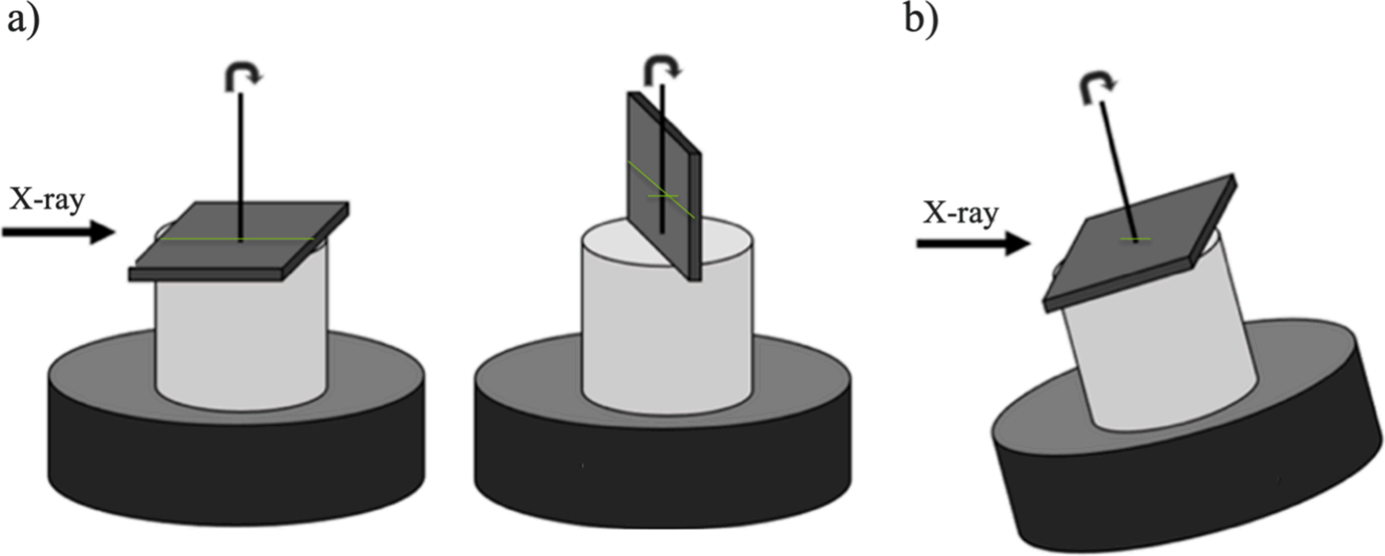
Comparison of imaging geometries for planar samples: (*a*) standard tomography geometry with placing the sample horizontally and vertically, (*b*) laminography geometry. Green lines represent possible X-ray propagation paths inside the sample.

A possible strategy for a more efficient acquisition of TXM data is the laminography scanning technique (Helfen *et al.*, 2005 ▸; Helfen *et al.*, 2007 ▸; Nikitin *et al.*, 2024 ▸). A comparison between tomography and laminography scanning of planar samples is schematically demonstrated in Fig. 1 ▸. In standard tomography, the X-ray beam is blocked by the sample at certain angles, limiting the angular coverage. Laminography involves tilting the rotary stage by 20–30° along the beam direction, allowing the scanning of planar and highly X-ray absorbing samples with improved image quality and reduced radiation damage. In particular, while standard tomographic imaging imposes sample thickness constraints in two dimensions (*e.g.* 30–50 µm), laminography relaxes this requirement to only one dimension.

Laminography imaging has been demonstrated at several synchrotron facilities and laboratory-based CT systems worldwide [see Fisher *et al.* (2019 ▸) and Nikitin *et al.* (2024 ▸) and references therein]. A notable recent advancement is the development of the ptycho-laminography instrument at the cSAXS beamline of the Swiss Light Source (Holler *et al.*, 2019 ▸; Holler *et al.*, 2020 ▸), which enables efficient imaging of integrated circuits at sub-20 nm resolution. In our previous work (Nikitin *et al.*, 2024 ▸) we provided a detailed overview of laminography instruments worldwide and also described the implementation of laminography at the micro-CT beamline 2-BM of the APS, including a fast reconstruction algorithm developed as part of the *TomocuPy* software package (Nikitin, 2023 ▸). Moreover, we demonstrated how laminography can be used to minimize the need for cutting procedures when scanning large samples by employing mosaic scanning protocols, which involve scanning different regions of the sample and stitching the projection data.

In this work, we present our laminography implementation at the TXM of beamline 32-ID of the APS. We will also describe the modifications made to the instrument following the recent upgrade of the APS (APS-U) to a fourth-generation synchrotron and present initial imaging results. Laminography-based TXM imaging is demonstrated on an integrated circuit sample and on high-strength magnesium alloy (WE43) powder particles (Luxfer Magtech, USA) distributed over a flat, tilted substrate, allowing a selected particle of interest to be imaged efficiently. Finally, we will briefly outline future enhancements and potential applications of the developed instrument for scanning large millimetre-size samples and efficient, dynamic 3D nano-imaging.

## 2. Laminography TXM setup at beamline 32-ID

The TXM at station 32-ID-C of the APS has been in operation and continuously improved since 2015 (De Andrade *et al.*, 2016 ▸; De Andrade *et al.*, 2021 ▸). It was temporarily shut down during the APS-U, which was completed in 2025. In the following, we describe the changes made to the instrument and introduce its new laminography capabilities.

### 2.1. TXM after APS Upgrade

**Figure 2:**
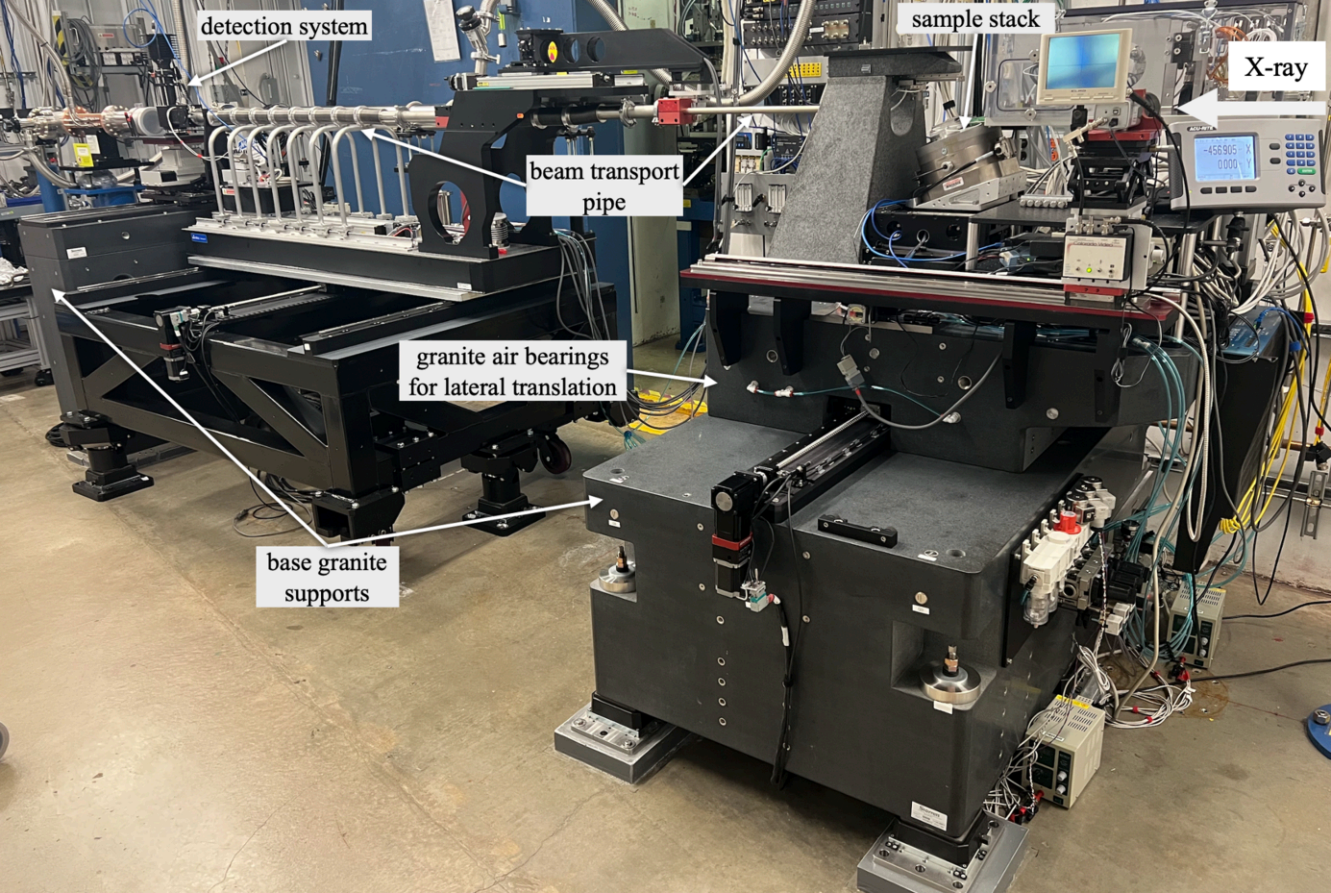
Overview of the upgraded TXM with lateral translation at station 32-ID-B of APS.

During the APS upgrade, the TXM was moved from station 32-ID-C located at 70 m from the source to station 32-ID-B located at 35 m from the source. The instrument base support was completely redesigned. It now incorporates an additional granite air-bearing stage, allowing the entire instrument to be moved in and out of the beam, see Fig. 2 ▸. This design allows full access to the downstream station C even while the TXM is operating in the upstream station B, enabling setup and preparation of complex high-speed imaging experiments (another program at beamline 32-ID) — which typically require long setup times but very short measurement durations. Once the high-speed setup is ready, a rapid switchover is achieved by simply moving the TXM out of the beam path. The whole change takes about 1 h, which significantly improves the overall beamline utilization efficiency.

**Figure 3:**
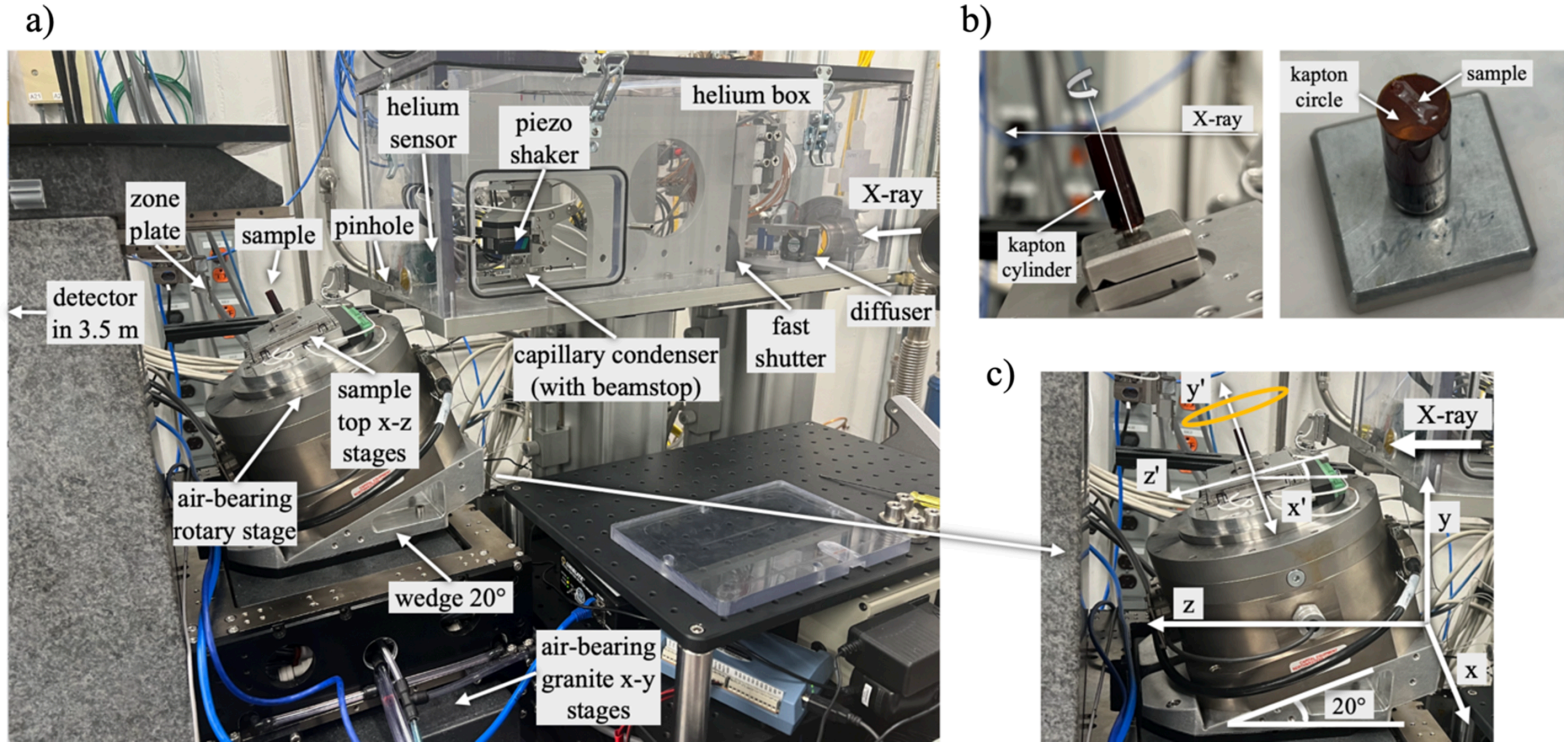
(*a*) Laminography TXM setup at beamline 32-ID of APS; (*b*) an example of sample mounting for laminography; (*c*) schematic showing coordinate systems used for alignment of the rotary stage and samples.

Our beamline operates with a 2.1 m-long, 28 mm-period planar hybrid permanent magnet undulator and a Si(111) double-crystal monochromator (Kohzu Precision Co., Japan). When used with the TXM, the monochromator operates over an energy range of 6.5–12 keV with a relative bandwidth of Δ*E*/*E* ≃ 1.3 × 10^−4^. Due to the small source size and low beam divergence, the new setup does not require additional beam collimation with compound refractive lenses (Snigirev *et al.*, 1996 ▸) as was done prior to the APS-U (De Andrade *et al.*, 2021 ▸). The beam, after the monochromator, propagates directly to the box where the capillary condenser (Sigray, Inc., USA) is hosted, see Fig. 3 ▸(*a*). The box is filled with helium to reduce X-ray absorption by air. The level of helium is controlled using a proportional valve and helium sensor. Ahead of the condenser, the beam coherence is reduced using a rotating diffuser. A fast shutter (Vincent Associates, USA) is placed after the diffuser to enable efficient, automated collection of dark- and flat-field during tomography scanning.

### 2.2. X-ray illumination and optical configuration

The TXM at beamline 32-ID provides two imaging configurations. The first is a lower-resolution setup that uses a Fresnel zone plate (FZP) with a 30 nm outer zone width (Δ*r*_*N*_), paired with a Sigray condenser with numerical aperture (NA) matching that of the FZP to ensure proper illumination. The second is a higher-resolution configuration employing an FZP with a 16 nm outermost zone width and a Sigray condenser having a higher NA. For simplicity, this work focuses on demonstrating laminography imaging using the lower-resolution setup.

**Figure 4:**
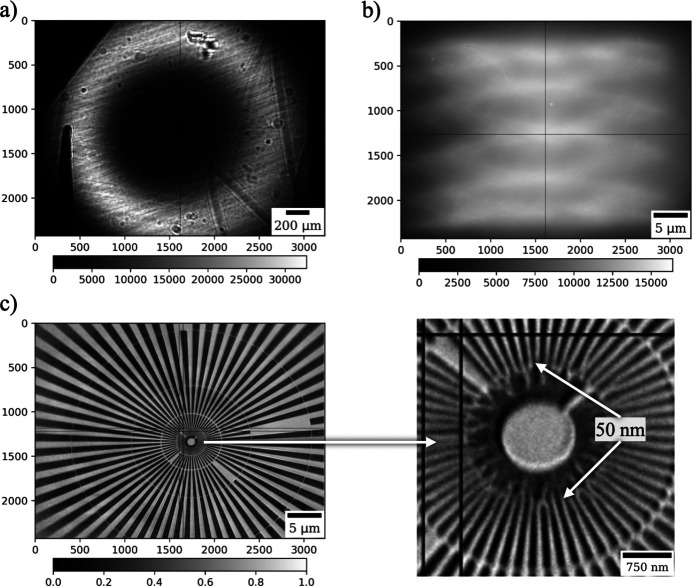
Image on the detector in the micro-CT regime without TXM optics (*a*), and in the nano-CT regime with TXM optics (*b*); a flat-field corrected image of the Siemens star test pattern in the TXM regime (*c*).

The capillary condenser focusing the beam to the sample in this setup has inner and outer diameters of 475 µm and 749 µm, respectively. It is efficiently illuminated by a monochromatic beam. In our setup we set the monochromator crystals to reflect 8 keV X-rays and we set the undulator gap such that the nominal energy of the first undulator harmonic is 8.05 keV. This 50 eV detuning from the peak of the harmonic allows us to make use of the off-axis undulator radiation which has a ‘donut’-shaped intensity profile [Fig. 4 ▸(*a*)]. With such a shape the beam illumination is used more efficiently since losses due to the required beam stop located at the entrance and centered in the middle of the condenser are minimized. It should be noted that shaping the beam into a ‘donut’ became possible only after the APS upgrade to the fourth-generation source.

The hollow cone beam from the condenser illuminates the sample with a focal spot size of about 2–3 µm at the sample plane. The unwanted portion of the beam is blocked using a pinhole placed outside of the helium box, see Fig. 3 ▸(*a*). The image is formed on a detector positioned 3.5 m downstream of the sample, using an in-house-fabricated FZP with a 30 nm outermost zone width (Gleber *et al.*, 2014 ▸). The condenser is characterized by a NA of 2.45 × 10^−3^, which is close to the 2.58 × 10^−3^ NA of the FZP at 8 keV, ensuring an optimal illumination of the objective lens. Distances between the sample and optical elements are large enough to accommodate the installation of environmental cells: the distance between the condenser exit plane and the sample plane is 96.9 mm; and that between the sample and zone plate is 59 mm. The illumination field at the sample location is controlled with a three-axis piezo (Piezosystem Jena, Germany) stage that ‘shakes’ the condenser in the transverse plane following a defined Lissajous trajectory at high frequency. The frequency of completing one Lissajous cycle is chosen with respect to the exposure time for one projection. In our setup, we were able to shake the condenser to illuminate the whole field of view on the detector at a speed of more than 50 Lissajous trajectories per second, which allowed us to work with less than 0.02 s exposure time per projection. An example of the illumination field on the detector with all TXM optics in place is shown in Fig. 4 ▸(*b*).

The visible light optics consists of a 50 µm GAGG+ scintillator (Crytur, Czech Republic) coupled with a 10× objective with NA of 0.42 (Mitutoyo Plan Apo Infinity Corrected series, Japan) and a SWTLU-C tube lens (Olympus, Japan). In the classic monochromatic configuration (Koch *et al.*, 1998 ▸) this gives a nominal magnification of 9×. The detector is an Oryx 31.0 MP Mono 10 GigE (Teledyne Vision Solutions, USA) with 6464 × 4852 pixel array and 3.45 µm pixel size (IMX342: Sony Semiconductor Solutions, Japan) which exhibits outstanding photometric performance (https://www.teledynevisionsolutions.com/learn/learning-center/machine-vision/emva-1288-overview-imaging-performance/). This sensor also utilizes a global shutter, which is advantageous because the integration of the shaken condenser is simpler and robust because it does not need to be precisely synchronized with a rolling shutter readout. The resulting pixel size, with the 2 × 2 binning mode and TXM + objective magnification, is 13.2 nm. This corresponds to 1/2.3 of the outermost zone width of the FZP to satisfy the Nyquist sampling condition to prevent aliasing artifacts.

### 2.3. Sample stack

The rigid sample stack has not been significantly modified to accommodate the laminography setup, see Fig. 3 ▸(*a*). As before the APS-U, precise vertical positioning and alignment of the rotation axis with respect to the beam are achieved using *x*–*y* granite air-bearing stages. Once the desired positions are set, the air supply is turned off, and the stage effectively functions as a rigid monolithic block, maximizing the thermal and mechanical stability of the system. Provided there is no drift, the high-precision air-bearing rotary stage (Professional Instrument Company, USA) maintains alignment with exceptional stability and low runout error (<20 nm). Precision piezo *x*–*z* stages (SmarAct, Germany) are mounted on top of the rotary stage for reliable sample alignment and have a uni-directional repeatability of <80 nm.

The resolution of the resulting TXM instrument was evaluated using a setup with an FZP having a 30 nm outermost zone width. In Fig. 4 ▸(*c*) we show an image of the Siemens star test pattern containing 50 nm features. The displayed image was acquired with 0.1 s exposure time and flat-field-corrected to easily observe the small features.

### 2.4. Adjustments for laminography

The only change to enable laminography TXM measurements is adding a 20° wedge under the rotary stage. The wedge was precision machined (AJR Industries Inc., USA) from aluminium. Notably, the wedge is placed such that the rotary stage is tilted downstream rather than upstream. This is needed for more radiation efficient scanning of samples, as will be described below.

The 20° wedge angle was chosen based on simulation results and experience of reconstructing experimental micro-computed laminography data from beamline 2-BM of the APS. Larger angles typically introduce significant laminography artifacts due to the missing cone issue in the frequency domain [see Nikitin *et al.* (2024 ▸) for details], while smaller angles yield significant beam attenuation (blockage) for planar samples.

We propose a simple sample mounting protocol for nano-laminography, shown in Fig. 3 ▸(*b*). The protocol involves using a Kapton cylinder and Kapton disks (American Durafilm, USA). A Kapton cylinder of diameter 8 mm is attached to a regular kinematic mount typically used for mounting pins in regular TXM nano-tomography imaging. The cylinder has a height of about 10–15 mm and wall thickness of 0.23 mm. Based on our tests, such wall thickness yields about 80% transmission of X-rays at 8 keV and ensures enough rigidity to perform measurements with 30–60 nm resolution. Samples are placed on a Kapton disk, which is then attached to the top part of a Kapton cylinder. For large flat samples, adhesive is applied only to the edges to ensure that X-rays do not pass through the glued regions during laminography scanning. This approach minimizes resin degradation and prevents sample movement caused by radiation-induced adhesive failure. Small, powder-like samples typically do not need to be fixed, as static forces are usually sufficient to keep the powder particles in place. It has been found that in some cases it is beneficial to glue the Kapton disk to the cylinder upside down to effectively encapsulate the sample and thus prevent possible effects from the environment (*e.g.* air flows inside the end-station).

For a radiation-dose-efficient approach, it is now clear that the rotary stage needs to be tilted in the downstream beam direction since in this case the beam first hits the Kapton cylinder wall (losing the photon flux) and then illuminates the sample. If the rotary stage is tilted upstream then the sample receives more radiation dose and heat load, while the image on the detector is not affected. This is particularly important when working with higher resolution imaging (10–20 nm) where more rigid sample holders are required for stability. Based on our experiments, laminography with higher resolution (*e.g.* with the 16 nm outermost zone width FZP objective) suffers from insufficient rigidity of the Kapton cylinder. We observe motion artifacts in reconstructions in this case. The problem was resolved by replacing the Kapton cylinder with a glassy carbon crucible (HTW, Germany). These crucibles are very rigid and radiation-hard; however, the commercially available versions have a minimum wall thickness of 0.5 mm, which reduces the X-ray flux by at least 50%. In such cases, it is crucial to first propagate the beam through the crucible wall and then through the sample.

### 2.5. Data acquisition and reconstruction

Data acquisition in the laminography geometry has several differences compared with conventional tomography. The first is the alignment procedure for the rotary stage. In the wedge-based setup, it is necessary to verify the exact roll and pitch angles of the rotary stage, which correspond to tilts around the *z*- and *x*-axes, respectively, as shown in Fig. 3 ▸(*c*). As a test sample for such alignment, it is common to use a tungsten pin, where the tip of the pin acts as a representative feature.

The roll angle can be adjusted by rotating the detector. In tomography, this is typically done by placing the feature (tip of the pin) at the left or right border of the detector field of view when the rotary stage is at 0°, and comparing the vertical coordinate of this feature when the rotary stage is at 180°. Due to the tilted geometry, this procedure is not sufficient for the roll angle adjustment. An additional step required for laminography is to ensure that the sample top-*x* stage [moving along the *x*′-direction shown in Fig. 3 ▸(*c*)] translates the object truly perpendicular to the beam, *i.e.* along the *x*-axis shown in the same figure. This property is not required for tomography and is generally not verified at a beamline. To verify this property, we translate the feature using the top-*z* stage [moving along the *z*′-direction shown in Fig. 3 ▸(*c*)]. Notably, in this case the image of the feature on the detector will move vertically, since we are in the laminography geometry. We then fine-tune the rotation angle to ensure that the image of the feature on the detector does not shift horizontally during this translation. Once this condition is met, we reset (reassign the angle without rotation) the rotary stage position to 90°. We then return to 0° and adjust the roll angle, as is typically done in standard tomography.

We are not aware of any quick and accurate way to check the pitch angle for the rotary stage. With our instrument we check it during reconstruction, as described by Nikitin *et al.* (2024 ▸). For that, reconstruction is performed using different pitch angles and the proper angle is chosen based on the result with the fewest artifacts at the borders. The procedure is carried out only once with a stable high-contrast sample. In principle, the roll angle can also be determined by analyzing reconstructions. However, in this case one may end up having to simultaneously evaluate artifacts caused by incorrect pitch, roll, and rotation axis alignment, which we have found to be impractical.

The sample alignment procedure in nano-laminography with TXM is particularly challenging due to the presence of two rotational axes. If the alignment is incorrect, the object’s image on the detector follows an elliptical trajectory during rotation, corresponding to the 2D projection of the orange ellipse shown in Fig. 3 ▸(*c*) onto the downstream detector. In this trajectory, the directions of the semi-axes correspond to the two rotation axes, vertical and horizontal, while their relative lengths are determined by the distance between the reference point on the sample and the system’s pivot point.

In our instrument, the sample alignment is generally performed in two steps. First, a coarse alignment is carried out in the micro-resolution regime, which is possible when all optical components (condenser, FZP, and pinhole) are kept outside the beam path. Once the region of interest is aligned and coincides with the pivot of the system, the second alignment step is carried out in the nano-resolution (TXM) regime. This involves an iterative procedure acting on multiple degrees of freedom. The goal is to keep the region of interest centered in the field of view and in focus while slowly rotating the sample. The procedure involves using all stages in the sample stack: *x*–*y* granite, *x*–*z* sample top, and the rotary.

For laminography scanning we use *TomoScan* (https://tomoscan.readthedocs.io/), a Python module for collecting computed tomography (and now laminography) data at the APS. A typical laminography scan requires collecting projections over 360° as opposed to regular tomography requiring a 180° interval. Each scan involves collecting dark fields, flat fields, and projections in the fly scan mode. All raw data are captured to an HDF5 (Hierarchical Data Format version 5) file using a plugin of *EPICS* (*Experimental Physics and Industrial Control System*) *AreaDetector* (Rivers *et al.*, 2010 ▸). Following the DXfile data template (De Carlo *et al.*, 2014 ▸), the created HDF5 file also contains metadata which includes all scan parameters and beamline settings (positions of motors, energy, ring current, *etc*.). DXfile metadata can be easily printed and validated using *meta-cli* (https://github.com/xray-imaging/meta-cli). At the end of each scan, all TXM elements are moved out of the X-ray path and one projection is collected in the micro-resolution regime and also stored in the HDF5 file. The projection image is a useful reference to have for keeping track of the scanned regions of interest and will be part of the experiment electronic log (https://tomologcli.readthedocs.io).

Laminography data reconstruction is performed using *TomocuPy* (Nikitin, 2023 ▸), more specifically its extension for laminography data processing (Nikitin *et al.*, 2024 ▸). *TomocuPy* is a Python package and a command-line interface for reconstruction of tomographic/laminographic data. Most processing operations are implemented on GPU (graphics processing unit). Laminography reconstruction with *TomocuPy* consists of three steps: (1) manual rotation axis search by examining reconstructions of one slice for different rotation axes, (2) manual pitch angle search by examining reconstructions of one slice for different pitch angles, (3) full reconstruction of the volume using the found rotation axis and pitch angle.

## 3. Results

To demonstrate the developed laminography TXM capability we conducted two nano-laminography measurements at beamline 32-ID of the APS. The first set of measurements highlights how laminography simplifies sample mounting when scanning a regularly shaped specimen. By bringing the sample of interest onto the rotation axis, it can be easily selected from a large set deposited on the laminography mounting surface. The second series of measurements show a general application of laminography for scanning planar samples having large sizes in lateral dimensions.

### 3.1. Magnesium alloy powder particle

The first measurements presented are the isolation of a single high-strength magnesium alloy (WE43) powder particle with a spherical morphology among a dispersion of particles. Diameters of the particles are ranging between 15 and 53 µm. The alloy is commonly used in aerospace, automotive, and biomedical applications due to its high strength-to-weight ratio, corrosion resistance, and bio-compatibility. It consists of magnesium (93–95%), yttrium (4%), neodymium (2–3%), zirconium (0.5%) and other elements with lower concentration. Yttrium, neodymium and even zirconium offer significantly higher contrast relative to magnesium, facilitating detection of solute rich segregate phases.

**Figure 5:**
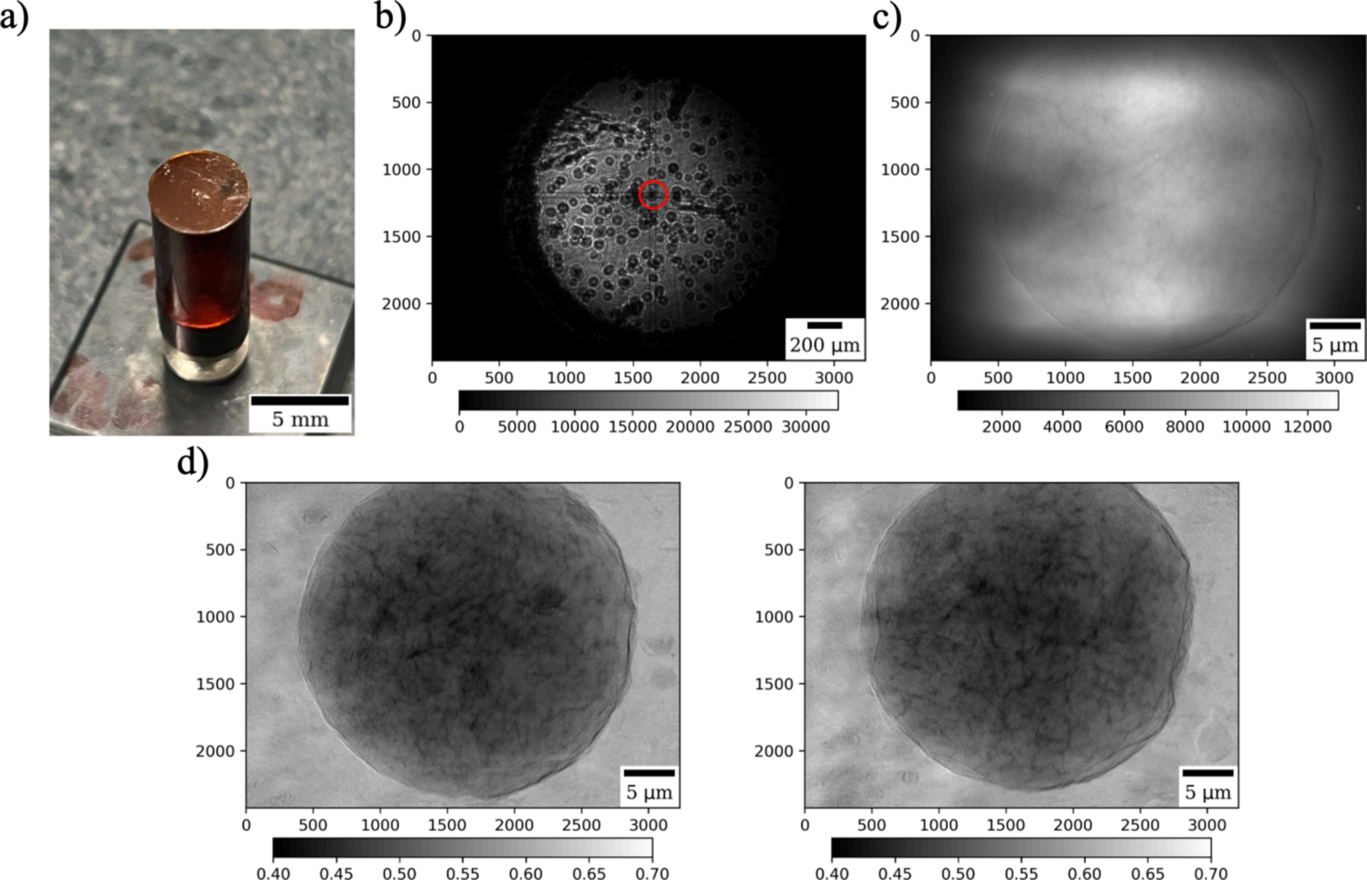
Laminography TXM imaging of high-strength magnesium alloy (WE43) particles spread on a Kapton tape: (*a*) powder particles on a Kapton tape fixed to a Kapton cylinder, (*b*) selection of a particle in the micro-resolution regime, (*c*) zooming to the particle by placing the TXM optics in place, (*d*) projections after flat-field correction at 0 and 90° rotation.

We sprinkled the powder onto a 8 mm Kapton disk and fixed the disk onto the Kapton cylinder, see Fig. 5 ▸(*a*). The particles were not fixed to the Kapton disk since static forces were sufficient to keep the particles in place. A kinematic mount was then placed on the rotary stage and checked in the micro-resolution regime, see Fig. 5 ▸(*b*). One can see many particles that can be sequentially imaged with TXM one after another. Note the pronounced intensity oscillations at the particle boundaries, resulting from interference of coherent X-ray waves after propagating over 3.5 m between the sample and the detector. We selected a random particle by positioning it in a way that it rotates at the center (red circle) in the figure. When alignment is completed in the micro-resolution regime we moved all TXM optics in place and repeated alignment at nano-resolution. Fig. 5 ▸(*c*) shows a projection of the particles in the TXM mode. A spherical shape of the particle is now clearly visible, filling the entire field of view. Small features inside the sample are observable in projections after flat-field correction, see Fig. 5 ▸(*d*).

Laminography data acquisition for the WE43 alloy particle involved collecting 1440 angular projections over 360° angles, with 0.1 s exposure time per projection. Total acquisition time including collection of dark and flat fields was about 2.1 min. We note that the number of collected projection angles is lower than the required number of angles to satisfy the Nyquist criteria for resolving each pixel. This criterion can be relaxed since a pixel size of 13.2 nm in these measurements is smaller than the 30 nm outermost zone width of the FZP. Moreover, our goals in this work are not to demonstrate ultimate instrument resolution or speed but rather show advantages of the nano-laminography technique in general.

**Figure 6:**
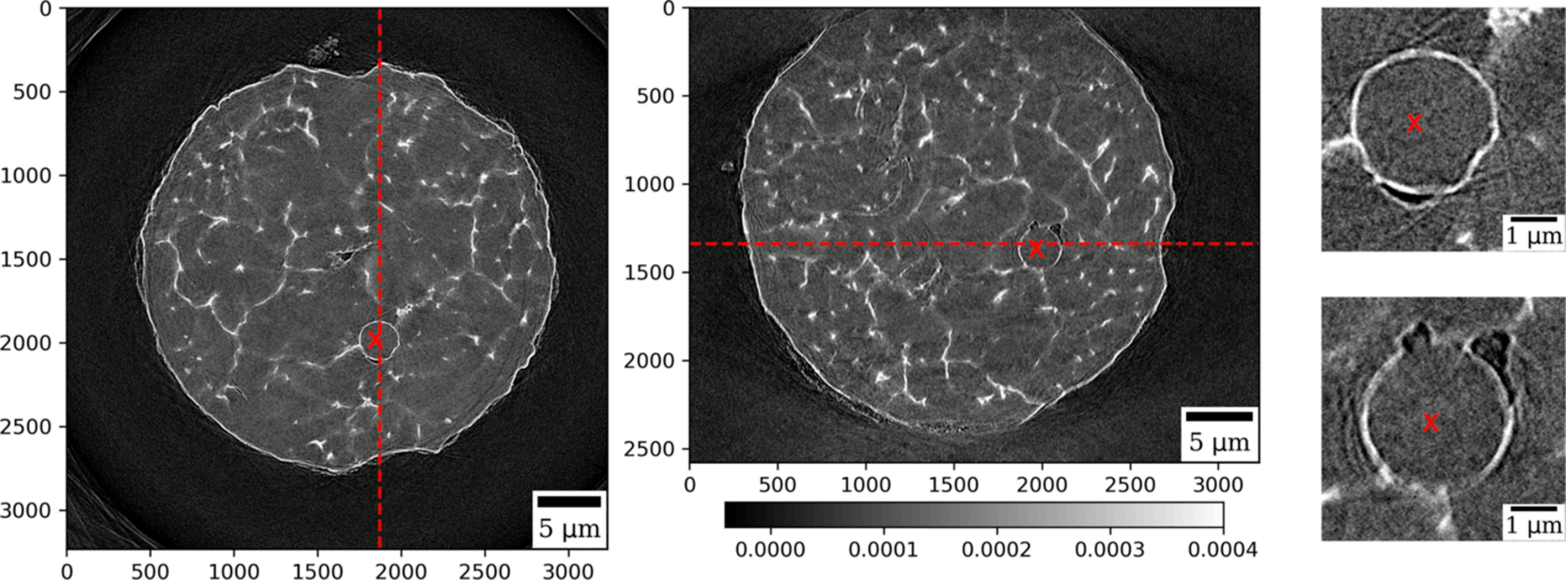
Reconstruction of the WE43 alloy particle: horizontal (left) and vertical (middle) slices through the reconstructed volume, and zoomed-in region marked with red crosses (right). Red dashed lines indicate positions of the slices with respect to each other.

In Fig. 6 ▸ we demonstrate reconstruction results for the measured WE43 alloy particle. The figure shows horizontal and vertical slices through the particle and zoomed regions showing high-resolution features. The gray color regions correspond to magnesium, while regions of brighter color contain different concentrations of yttrium and neodymium. In the zoomed-in regions, we can also observe porosity in this gas atomized particle (darker regions).

### 3.2. Integrated circuit

The second set of measurements features an integrated circuit with dimensions of 2 mm × 2 mm × 0.2 mm. The circuit primarily comprises a silicon substrate, with high-density elements such as transistors and interconnects integrated on its surface. Notably, only the 20 µm out of 200 µm thickness contains the active region where these components are located. More information about X-ray imaging of microelectronics have been given, for instance, by Aidukas *et al.* (2024 ▸).

**Figure 7:**
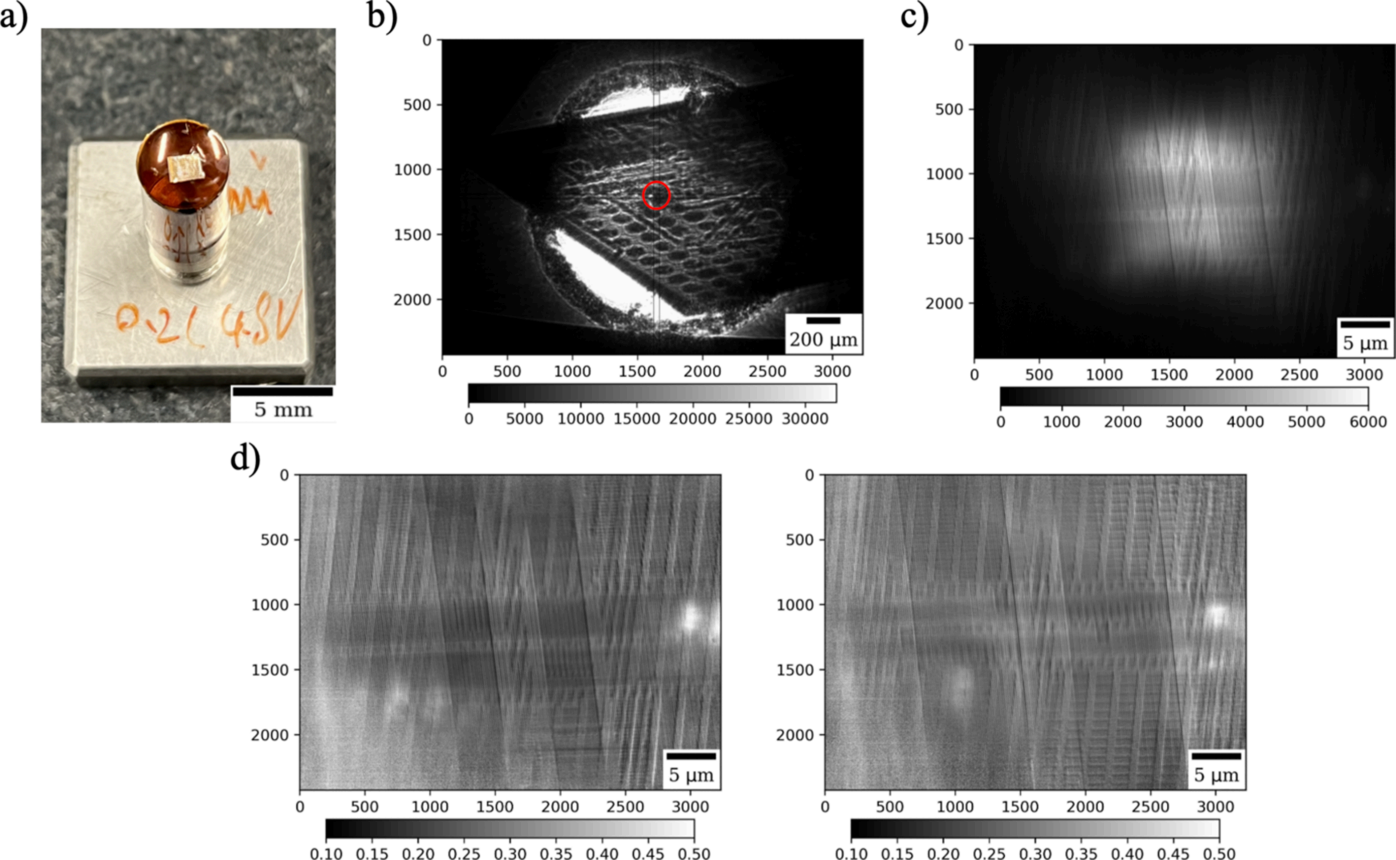
Laminography TXM imaging of an integrated circuit: (*a*) integrated circuit fixed to a Kapton tape, (*b*) selection of a region of interest in the micro-resolution regime, (*c*) zooming to the region of interest by placing the TXM optics in, (*d*) projections after flat-field correction at 0 and 90° rotation.

The integrated circuit was fixed to a Kapton disk from one side with UV resin (Bondic, Canada), see Fig. 7 ▸(*a*). The region with the resin is not illuminated during laminography scanning to prevent melting/degradation of the resin and consequent sample motion artifacts. The kinematic mount was then placed on the rotary stage and checked in the micro-resolution regime, see Fig. 7 ▸(*b*). For scanning, we selected a region of interest containing an intersection of straight line features. Fig. 7 ▸(*c*) shows a projection through the sample in the TXM regime. Small features inside the sample are observable in projections after flat-field correction, see Fig. 7 ▸(*d*). It should be noted that image quality is reduced in areas with insufficient illumination.

Fig. 7 ▸(*c*) also demonstrates the flexibility of choosing the illuminated area on the sample which is practically realized by reducing the amplitudes of the Lissajous trajectory of the shaken condenser. In the figure, the beam illuminates only the central part of the integrated circuit (20 µm × 20 µm). The circuit is periodic and for the preliminary measurements we report here it is enough to scan a small region of it allowing an increased projection acquisition rate to be used. Increasing the illumination area will reduce the photon flux per pixel on the detector, leading to longer exposure times to keep an acceptable image quality. Such flexibility is also beneficial when scanning samples smaller than the field of view, as directing all photons onto the sample can reduce acquisition time.

Laminography data acquisition for the integrated circuit with the reduced illumination area involved collecting of 1200 projections over 360° angles, with 0.05 s exposure time per projection. The total acquisition time in this case was about 1 min.

**Figure 8:**
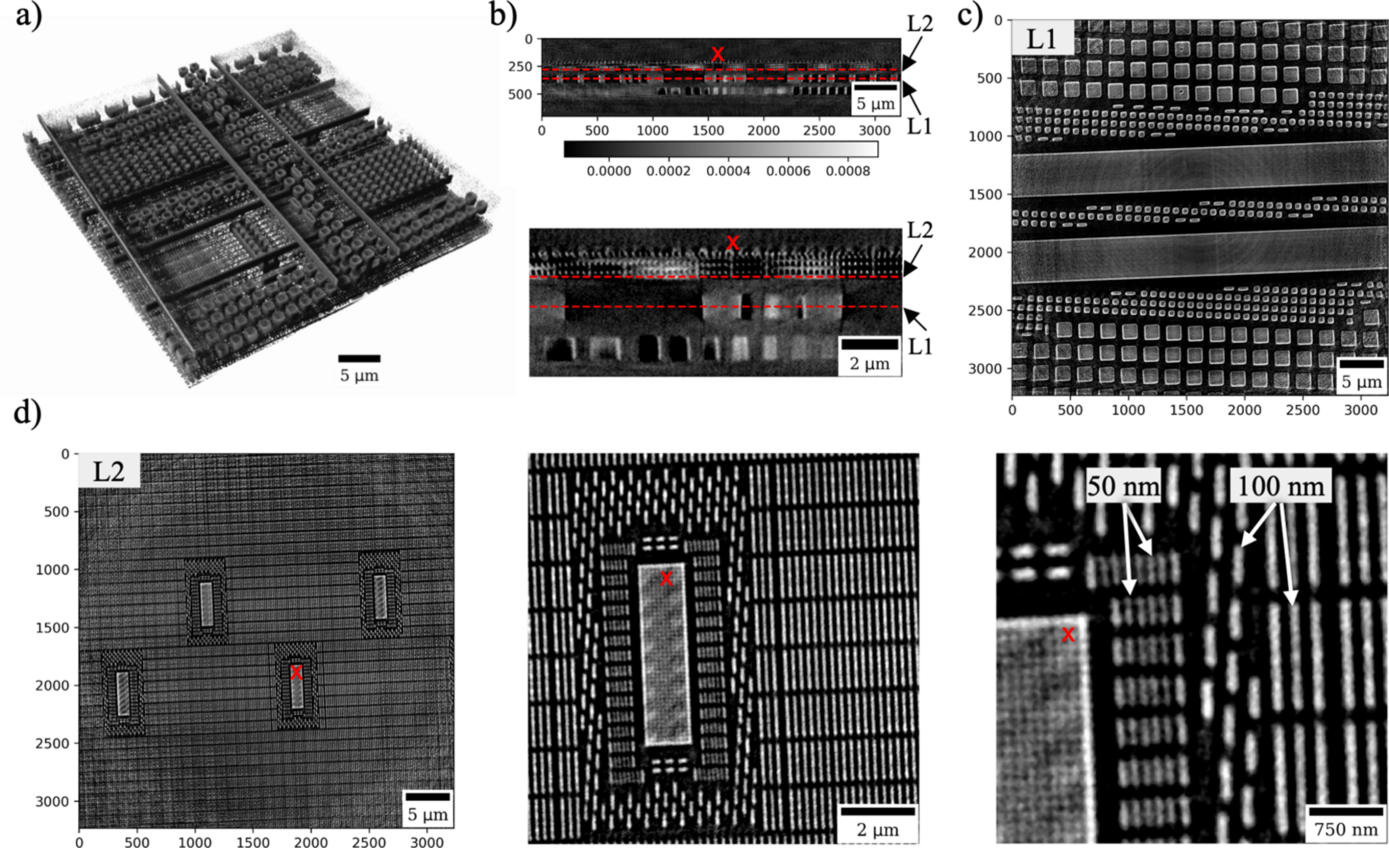
Reconstruction of the integrated circuit sample: (*a*) 3D rendering of the reconstructed volume, (*b*) vertical slice through reconstruction with zooming in and marking positions of demonstrated horizontal slices, (*c*) horizontal slice through the reconstructed volume at layer L1, (*d*) horizontal slice through the reconstructed volume at layer L2 and zoomed-in regions. Red crosses indicate positions of the zoomed-in regions.

In Fig. 8 ▸ we demonstrate reconstruction results for nano-laminography imaging of the integrated circuit. A 3D rendering of the reconstructed sample is shown in panel (*a*), and a video animation of the rendering is provided in the supporting information. Panel (*b*) shows a vertical slice through the middle of the sample, and its zoomed version for better visibility of features. The red dashed lines show positions of demonstrated horizontal slices marked as layers L1 and L2. Layer L1, containing only large features (300–1000 µm), is shown in panel (*c*). In contrast, Layer L2 in panel (*d*) reveals finer structural details. The bottom right part of the figure clearly highlights 50 nm and 100 nm features.

## 4. Conclusions and outlook

The first nano-laminography imaging using the transmission X-ray microscope has been successfully demonstrated at beamline 32-ID of the Advanced Photon Source. This technique enables efficient scanning of planar samples and simplifies sample mounting, thereby improving experimental throughput. Thanks to the upgraded APS source and instrument components, full 3D scans of 40 µm-thick samples at 13 nm voxel size can now be completed within a few minutes, which is a 5–10× improvement in scan speed compared with the TXM before the APS-U (*cf*. De Andrade *et al.*, 2016 ▸; De Andrade *et al.*, 2021 ▸). This acceleration was made possible by tailoring a ‘donut’-shaped illumination profile, which is feasible only with the upgraded source. The new illumination profile improved beam efficiency by minimizing losses caused by the central beam stop at the entrance of the condenser.

Initial TXM measurements after the APS-U were performed on a variety of samples, and results for several of them were reported in this paper. A spatial resolution of approximately 50 nm was confirmed using a 2D Siemens star test pattern and through 3D reconstruction of features within an integrated circuit sample. The demonstrated capability for rapid, high-resolution imaging of integrated circuits opens the door for future studies of their dynamic behavior under varying environmental conditions.

Additionally, we proposed a simple and efficient sample-mounting strategy tailored for laminography. It uses a Kapton disk and cylinder (or a glassy carbon crucible when higher stability is required). Powder-like particles can be distributed across the Kapton surface without the need for adhesives, as electrostatic forces are sufficient for immobilization. This avoids sample motion commonly introduced by glues, which can be affected by long X-ray exposure. We demonstrated this approach by imaging an alloy particle selected from a powder sample.

In the paper, we also detailed the key modifications made to the TXM during the APS-U, including relocating the instrument to an upstream station, mounting it on a movable granite base, and replacing the condenser. This information, along with photographs of the upgraded instrument, is included in the paper to help future beamline users efficiently plan their experiments. By understanding the new instrument geometry, users can better prepare for sample mounting and design potential *in situ* experiments involving complex environmental cells.

We note that the primary goal of this work was not to optimize resolution or scanning time with the TXM instrument but rather to demonstrate the significant potential of its new laminography capability. The upgraded TXM system at beamline 32-ID is still under development and requires further improvements. As noted previously, the instrument was relocated from station 32-ID-C to 32-ID-B. However, the 32-ID-B hutch is not yet fully optimized for nano-imaging. Planned upgrades include improving the stability of environmental conditions, such as reducing temperature variations within the hutch and minimizing vibrations from surrounding equipment, including vacuum pumps.

So far, we have tested the laminography setup with an FZP having a 30 nm outermost zone width and an NA-matching condenser for this FZP. Our second setup, configured for higher resolution TXM imaging, involves an FZP with a 16 nm outermost zone width and another condenser. We plan to test this setup once we reach stable environmental conditions inside the station. Additional improvements include the use of higher efficiency FZPs and, in general, a finer optimization of the optics. We expect to further improve both spatial and temporal resolution, aiming to achieve 10 nm nano-tomography within a few seconds.

Another area in need of improvement is the reliability and automation of sample alignment. Currently, data acquisition is significantly faster than the time required for manual sample alignment. Introducing semi-automated or fully automated alignment procedures would greatly increase throughput. We observed notable radiation and heat-induced damage in certain samples during alignment. Addressing this issue will require the development of new scanning strategies. For example, initial semi-automatic alignment could be performed using a low-intensity beam, which may be achieved by implementing movable filters upstream of the sample. Additionally, data acquisition could be limited to a subset of projection angles that provide sufficient information for reconstruction. Incorporating advanced reconstruction algorithms that account for radiation- or heat-induced sample deformation, such as those proposed by Nikitin *et al.* (2021 ▸), will further help to mitigate these effects and improve overall imaging quality.

**Figure 9:**
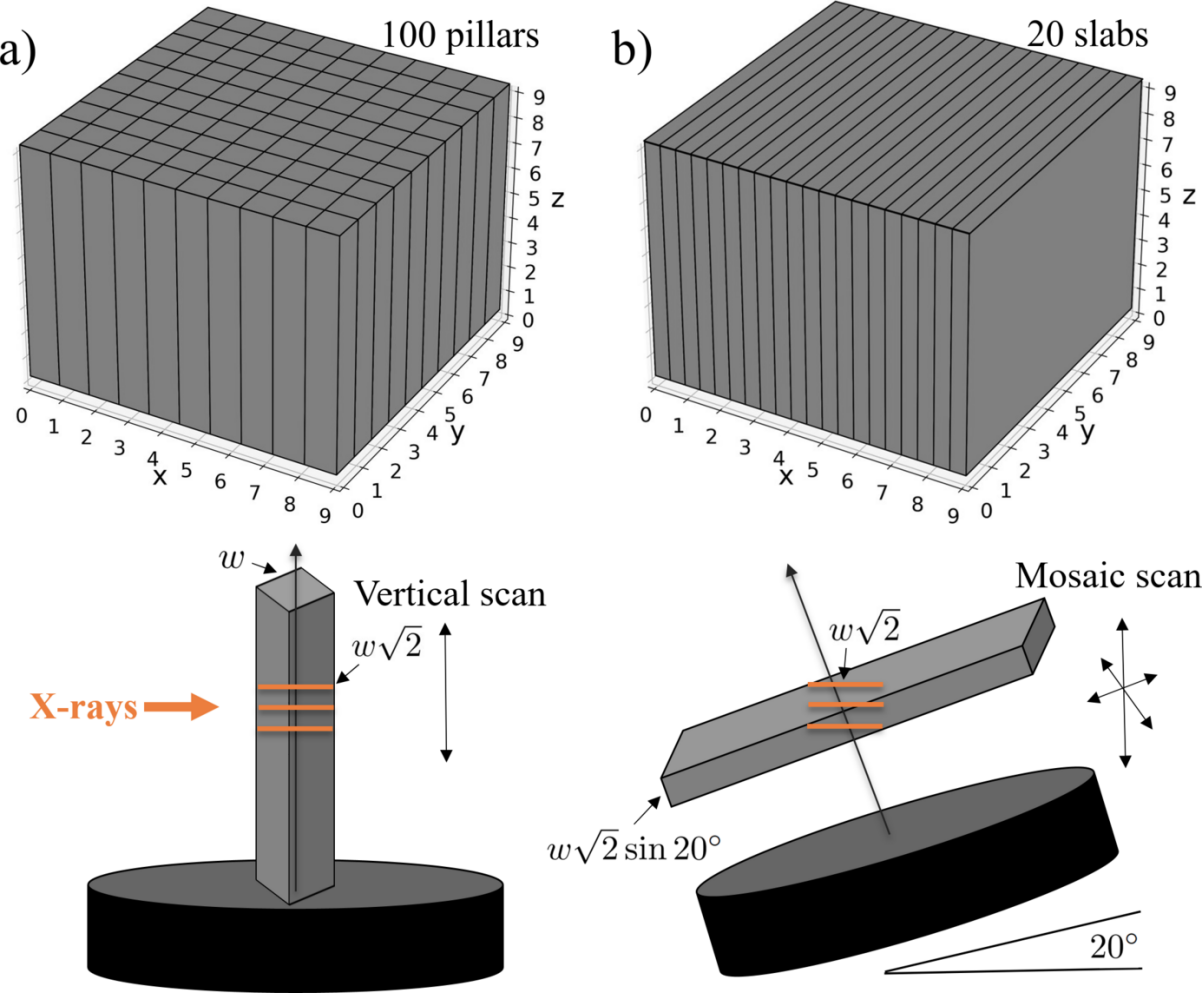
Schemes for scanning large samples by cutting them into parts with sufficient X-ray transmission: (*a*) tomography geometry with pillar-shaped parts, (*b*) laminography geometry with slab-shaped parts.

From a scientific standpoint, we plan to extend and adopt mosaic scanning strategies to image large, planar specimens already developed for micro-tomography (Nikitin *et al.*, 2024 ▸). With the mosaic scanning protocol the sample is scanned at different positions and projection data are stitched to form a large data volume for further reconstruction. This approach should allow for full 3D reconstruction of millimetre-sized, flat-shaped samples in nano-resolution. When applied to conventional large samples with several millimetre sizes in each dimension, the new laminography approach helps minimize the number of cutting procedures. Instead of cutting samples into many pillars and scanning them with regular TXM tomography, one can cut them into a significantly lower number of slabs and scan them in the mosaic laminography mode, see Fig. 9 ▸.

Expanding laminography TXM to enable dynamic imaging is also one of the primary goals for future studies. As mentioned earlier, the TXM at beamline 32-ID is a highly modular instrument capable of accommodating a variety of environmental cells. These experiments can benefit from the high spatial and temporal resolution offered by TXM at APS-U and the proposed laminography configuration. The laminography geometry is particularly well suited for dynamic experiments, as it imposes fewer constraints on sample size and allows *operando* devices to be connected from sample sides that are not illuminated by the X-ray beam. Potential applications include *operando* studies of battery materials, chemical and mechanical testing of microelectronics, and stress testing of materials.

## Supplementary Material

Volume rendering of the reconstructed integrated circuit sample. DOI: 10.1107/S1600577525007234/gy5078sup1.mp4
